# Computational and Experimental Studies on Combustion and Co-Combustion of Wood Pellets with Waste Glycerol

**DOI:** 10.3390/ma16227156

**Published:** 2023-11-14

**Authors:** Agnieszka Bala-Litwiniak, Dorota Musiał, Michał Nabiałczyk

**Affiliations:** Department of Production Management, Faculty of Production Engineering and Materials Technology, Czestochowa University of Technology, Armii Krajowej 19, 42-200 Czestochowa, Poland; dorota.musial@pcz.pl (D.M.); michal.nabialczyk@pcz.pl (M.N.)

**Keywords:** biomass, waste glycerol, combustion, domestic boiler, modeling

## Abstract

The shortage of fossil fuels and their rising prices, as well as the global demand for renewable energy and the reduction in greenhouse gas (GHG) emissions, result in an increased interest in the production of alternative biofuels, such as biodiesel or biomass pellets. In this study, the possibility of utilizing waste glycerol, as an addition to pine pellets intended for heating purposes, has been investigated. The usefulness of pellets containing glycerol additions has been compared in terms of applicable quality standards for wood pellets. The highest values of moisture (4.58%), ash (0.5%) and bulk density (650 kg/m^3^) were observed for pellets without glycerin waste. The addition of waste glycerol slightly increases the calorific value of the pellet (17.94 MJ/kg for 7.5% additive). A 10-kW domestic biomass boiler has been employed to burn the tested pellets. The consumption of analyzed fuels during boiler operation was determined. The concentration of CO, CO_2_ and NO_x_ in exhaust gases has also been examined. It was observed that the addition of 7.5% of waste glycerol contributes to the reduction in NO_x_ concentrations by 30 ppm and CO_2_ by 0.15%. The obtained experimental results were compared with the numerical calculations made with the use of ANSYS Chemkin-Pro. The conducted research indicates the legitimacy of utilizing waste glycerol as an additive to wood pellets. In addition, this type of addition has a positive effect on, among others, the increase in calorific value, as well as lower emissions of combustion products such as NO_x_ and CO_2_.

## 1. Introduction

The European Commission defined the Energy Strategy and the targets and scenarios for the period until 2030, as defined in the Framework for Climate and Energy. The Framework proposes a 40% cut in greenhouse gas (GHG) emissions, increasing the share of renewable energy sources by up to at least 27%, the continuous enhancement of energy efficiency, and the provision of competitive, affordable and secure energy [1,2]. In 2020, the European Green Deal declared to increase the level of greenhouse gas emission reduction to 55% compared to the values from 1990 [3,4,5]. The transport and energy sectors are the main anthropogenic sources in the European Union, responsible for emissions of over 20% and 60% of GHG, respectively, of which more than half is CO_2_ [6,7]. This is largely due to the fact that the sectors mentioned in most European countries are based on fossil fuels. The combustion of fuels such as coal, crude oil and natural gas causes significant emissions of pollutants, which, due to increasingly higher environmental protection standards, require continuous reduction [8,9].

There are many alternatives to fossil and conventional fuels, including solar energy, hydropower, geothermal energy, wind energy, hydrogen energy and biofuels [10,11,12]. Finding clean and renewable energy sources is one of the greatest challenges facing humanity in the medium and long-term perspective [13]. In accordance with Directive 2009/28/EC and the document “Draft National Policy Framework for the Development of Alternative Fuels Infrastructure”, in the transport sector, Member States were obliged to ensure a minimum 10% share of renewable energy by 2020, and by 2030, this share should increase to at least 14% [14,15,16]. Therefore, in recent years, there has been a significant increase in the production of liquid biofuels obtained from vegetable oils [17,18,19]. In European climatic conditions, the main raw material for biodiesel production is rapeseed oil, and less often, soybean, sunflower, corn or waste vegetable oils are used [20,21]. Biodiesel produced from rapeseed, called rapeseed methyl ester (RME), is produced in the transesterification process involving the methanolysis of rapeseed oil, which also produces waste glycerol [22,23,24]. The amounts of these undesirable wastes always exceed 10% of the weight of the obtained esters [25,26,27].

The dynamic increase in biodiesel production is therefore associated with the generation of large amounts of waste glycerol and the need for its disposal, which generates additional costs and thus affects the profitability of the process. So far, the most popular method of managing waste glycerol was its purification into pharmaceutical glycerin, which has numerous applications, including in the pharmaceutical, food and cosmetics industries [28,29]. In a number of studies published in recent years, there are many alternatives to using waste glycerol, including the transformation of glycerin into commonly used chemical compounds (production of hydrogen, ethanol and methanol, acrolein, organic acids and many others) [30,31,32,33,34,35]. Some authors also propose the use of glycerin as an additive to car fuels, animal feed or in the denitrification process in sewage treatment plants [36,37,38]. Unfortunately, the mentioned methods require preliminary cleaning, which involves additional costs. Due to the relatively high calorific value of this type of waste (16.1–22.6 MJ/kg depending on the raw material used to produce the biodiesel), using it as a fuel seems to be a reasonable solution [39].

Unfortunately, direct combustion of waste glycerol is problematic due to its high viscosity (at 22 °C about 110 cP) and relatively high ignition temperature (about 170 °C), which requires costly modifications to heating devices [40,41]. The best solution seems to be the co-combustion of waste glycerol with solid fuels such as biomass [42]. There is not much research on the use of glycerol as an additive to solid fuels. Zang and others [43] believe that the addition of glycerin waste to solid fuels could have a beneficial effect on reducing NO_x_ emissions as well as the formation of smaller amounts of ash. It was also observed that the addition of waste glycerol to biomass pellets reduces the bulk density and ash content and simultaneously increases the calorific value [44,45,46,47,48]. In turn, Potip et al. [49] attempted to co-incinerate waste glycerol with ground coffee and showed that with the increase in the addition of glycerin waste, the temperature and combustion rate increase, without affecting the exceedance of standards for exhaust gases such as CO, NO_2_ or SO_2_. Marrugo et al. [50] used waste glycerol as a binder in the production of pellets. They made pellets from sugarcane pomace, coffee husks and rice husks. The authors found that the addition of raw glycerin facilitates the binding of biomass particles during pellet production. Previous works have attempted to use waste glycerol as a binder during the production of wood pellets [26,42].

As mentioned earlier, the energy sector is responsible for over 60% of GHG emissions, therefore replacing fossil fuels with biomass is one of the cheaper and simpler solutions to reducing emissions not only of carbon dioxide but also of such toxic components of exhaust gases, such as sulfur dioxide, nitrogen oxides, carbon monoxide or chlorine compounds [51,52,53,54,55]. The main advantage of biomass is its production in the process of photosynthesis, where carbohydrates are obtained from CO_2_ and H_2_O under the influence of solar radiation. In this way, the CO_2_ obtained during combustion corresponds to the amount necessary for the production of biomass, resulting in a carbon dioxide emission of zero when burning biomass [10,56]. It is estimated that the amount of greenhouse gases generated in the biomass combustion process is lower by over 90% compared to coal combustion [57,58]. Therefore, the use of biomass for heating purposes (e.g., in the form of pellets) seems to be a simple, cheap and environmentally friendly alternative. The main source of biomass for the production of pellets is wood from products obtained from the wood industry, which in Poland results in higher costs for obtaining this type of pellet. This is due to the imposed VAT rate on wood fuels, which is 23%. For comparison, VAT on pellets from waste biomass is 8%. Therefore, the production of pellets from raw materials other than wood seems to be a more economical solution [39]. On the other hand, pellets made of wood have better physicochemical properties, including a higher calorific value [58,59]. An interesting solution may be the production of pellets with the addition of waste raw materials, e.g., the previously mentioned waste glycerol.

The aim of the research presented in this article was to determine the combustion conditions of wood pellets containing waste glycerol. The utilitarian goal was to produce wood pellets containing waste glycerol using a cheap and simple method and burn them effectively in a home heating boiler, while maintaining applicable emission standards [60,61,62]. This alternative can be cost-effective without the necessity of modifications of the heating devices. Glycerol co-combustion with the wood pellets can be a significant solution, because, besides the benefits of waste management, it also contributes to a reduction in harmful combustion products [39,42,63].

## 2. Research Methodology

### 2.1. Materials and Test Stand

The waste glycerol has been delivered from Trzebinia Refinery (RT S.A.), Trzebinia, Poland. The minimum content of glycerine in the waste was 80% wt, ash content was below 5% wt, MONG (Matter Organic Non Glycerol) was below 6% wt and the remaining was water. Calorific value of the waste was 22.6 MJ/kg. As the solid component, pine wood sawdust obtained from Opal-Drew Sawmill, Wapiennik, Poland, with fragmentation of 2–4 mm and a bulk density of 190 kg/m^3^ were used. Pellets were fabricated with the use of ZLSP 150B Pellet Mill with a power of 4 kW from Anyang Gemco Energy Machinery Co., Ltd., with factory situated in Anyang, Henan, China. The length and diameter of the pellets obtained were measured. In order to determine the selected physicochemical properties (ash content, moisture content, calorific value and CHN analysis), the analyzed pine–glycerol pellets were ground in a knife mill using a sieve matrix with a mesh size of up to 1 mm. The moisture content was determined based on a 1 g sample weight loss after drying at 105 ± 5 °C to a constant weight according to standard EN ISO 18134-3:2015 [64]. The ash content was determined by burning a 1 g sample of all the studied biomass fuels in a muffle furnace at 250 ± 10 °C for 50 min and then at 550 ± 10 °C for 4 h, according to standard EN ISO 18122:2015 [65]. The bulk density of the resulting pellets was determined in accordance with the EN ISO 17828:2016-02 standard [66]. A container with a volume of 0.002 m^3^ was used in the study. Several measurement tests were carried out and the obtained results were averaged. The calorific values of the analyzed fuels were determined using a KL-12 Mn calorimeter (PRECYZJA-BIT, Bydgoszcz, Poland) according to standard EN 14918:2009 [67]. The content of C, H, N for all four types of pellets was determined using an elemental analyzer (Truspec CHN628 LECO, St. Joseph, MI, USA). The simultaneous determination of the content of carbon, hydrogen and nitrogen was based on the Dumas method, also known as the high-temperature oxy-fuel combustion method. The content of the analyzed elements was determined using an infrared absorption detector (C and H) and a thermal conductivity detector (N). Based on the literature [37,63], the content of sulfur and chlorine in pine sawdust and glycerol does not exceed 0.01%. Therefore, these elements were omitted from the elemental composition analysis.

**Figure 1:**
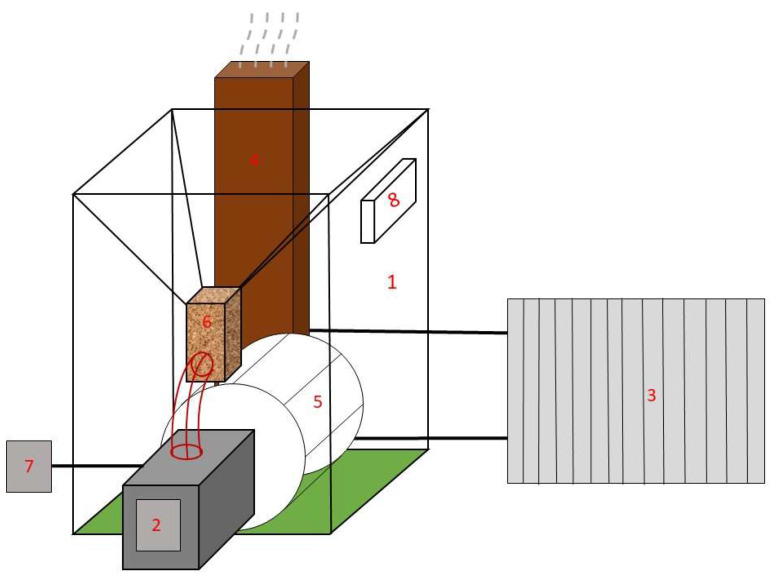
A simplified scheme of a test stand for combustion of biomass pellets, where 1—main boiler chamber; 2—burner; 3—heat exchanger; 4—funnel; 5—combustion chamber; 6—fuel feeder; 7—flue gas analyzer; 8—control panel.

A 10-kW domestic biomass boiler, Mini Bio type (Kostrzewa, Giżycko, Poland), was employed for pellet burning. The boiler was equipped with a burner adapted for the combustion of biomass pellets having a diameter of 6 to 8 mm. The chamber’s internal diameter was 0.32 m, and its total length was 0.48 m. The heat receiver was a radiator to which water flowed from the boiler water tank installed around the combustion chamber. The installation is equipped with a thermostat that keeps the water in the set temperature range. The temperature set point of the thermostat during pellet combustion was fixed at 60 °C. The temperature inside the combustion chamber was measured using a NiCr-Ni thermocouple. Depending on the distance from the burner, the temperature was in the range of 300–800 °C. The simplified scheme of the test stand is presented in Figure 1.

### 2.2. Methods

**Figure 2:**
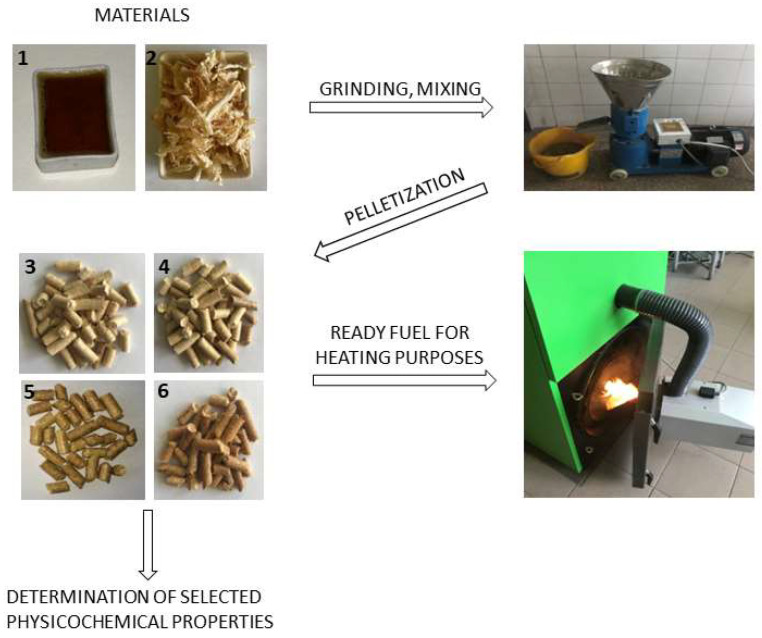
Schematic diagram of the methodology for the production of pine–glycerol pellets, where 1—glycerol; 2—pine sawdust; 3—PP; 4—PGP-2.5; 5—PGP-5; 6—PGP-7.5.

Four types of pellets were analyzed in the study: pine pellets without and with 2.5, 5 and 7.5% by weight share of waste glycerol. In the further part of the paper, the abbreviations “PP” for pine pellet and PP g for pine–glycerol pellet were used, respectively. The glycerol mass percentage in the pellet is specified at the end of the symbol (e.g., PP-G5 for pellets containing 5% wt of glycerol). The methodology for producing the analyzed pellets is shown in Figure 2.

During the combustion of each of the four types of pellets, a continuous chemical analysis of flue gases (CO_2_, CO and NO_x_) was carried out using the Vario-Plus analyzer (MRU, Poznań, Poland) utilized with infrared sensors. The analyzer probe (No. 7 in Figure 1) was placed approximately 10 cm from the burner. The value of the excess air coefficient measured by the analyzer was close to λ = 2.0 (± 0.5). Both temperature and flue gas concentrations were recorded continuously at the flue gas sampling point by the flue gas analyzer. The values were read every 2 min, on average. The burning experiments were repeated for all four analyzed fuels three times and special care was taken to maintain constant boiler operating conditions, including ambient temperature and thermostat temperature settings. The obtained experimental research results were compared with the numerical calculation using ANSYS Chemkin-Pro, ANSYS, Canonsburg, PA, USA.

The purpose of these considerations was to develop a numerical simulation procedure that would most reliably represent the chemical kinetics of the pine pellet combustion process. As suggested by previous research conducted by the authors, two combustion mechanisms were used for the calculations [59]:

- A large mechanism with 621 compounds and 27,829 chemical reactions developed by the CRECK Modeling Group [68,69];
- A small mechanism with 107 compounds and 652 chemical reactions developed by Glarborg et al. [70,71].

**Figure 3:**
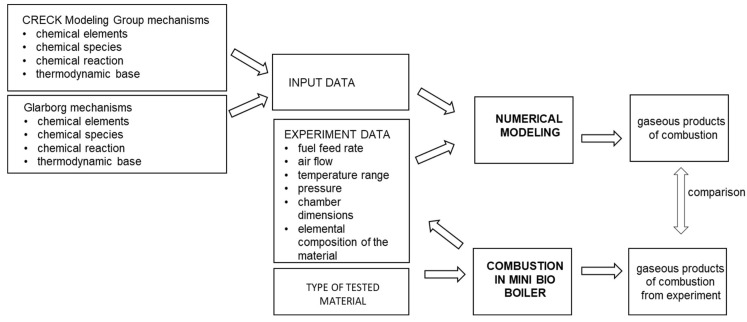
The scheme of the calculation procedure.

Both mechanisms contained the thermochemical and transport data necessary to perform the calculations. The diagram of the calculation procedure is shown in Figure 3.

The 0D steady state Perfectly Stirred Reactor (PSR) was used to conduct preliminary simulations. The selection of the chemical reactor was based on our previous works and that of other authors describing modeling processes for biomass combustion and co-combustion [59,72,73]. The following boundary conditions were adopted for the calculations:

- Fuel and air streams (Table 1);
- Elemental analysis of biomass (Table 2);
- Geometric parameters of the boiler;
- Temperature range of 300–850 °C;
- Residence time of 10 s.

The stream of supplied fuel and air, the elemental composition of the biomass and the temperature range in the combustion chamber were determined experimentally. The residence time was calculated based on the amount of exhaust gases leaving the combustion chamber and the chamber parameters. The obtained results of numerical simulations were reduced by the moisture content, and the shares of individual exhaust gas components were related to the dry exhaust gas content, so they could be compared with the experimental results (dry exhaust gas concentration from the analyzer).

## 3. Results and Discussion

### 3.1. Results of Experimental Studies

Table 1 shows the fuel and air consumption in the combustion process. The proximate and ultimate analyses, as well as the bulk density, length, diameter and calorific values of the analyzed pine–glycerol pellets are presented in Table 2.

Table 1 shows that the addition of glycerol increases fuel consumption which in turn contributes to an increase in the air flow. It results from Table 2 that the addition of glycerol contributes to an unsignificant reduction in pellet moisture. This is a rather unexpected result, since the applied glycerol contained ca. 10% of water, and it presumably results from the hygroscopic properties of glycerol. After the combustion of each type of analyzed fuel, the amounts of ash remaining were slightly lower for fuels containing 5% and 7.5% glycerol additions than for PP and PP-G2.5. A similar relationship was observed for bulk density. The trends observed in the above results are also confirmed by other research studies [44,45,47]. The calorific value of the waste glycerol (22.6 MJ/kg) is a little greater than that of pine sawdust utilized for pellet manufacturing (17.63 MJ/kg), and therefore, the increasing additions of glycerol to the pellets give rise to a slight increase in the calorific value.

The share of the waste glycerol in the cellulosic fuel does not significantly affect the quantitative changes of carbon, hydrogen and nitrogen in the final product. However, with the increase in glycerol content in the pellets, the small downward trend for the [C] and [N] and, at the same time, an upward trend for [H] and [O] are observed. This is due to the higher share of hydrogen and oxygen, lower share of carbon and lack of nitrogen in pure glycerin (C—39.13%, H—8.70%, O—52.17%). The produced pellets therefore meet the EN ISO-17225-2:2014 standard [74] in terms of moisture and ash content as well as diameter, length and bulk density. A much higher nitrogen content can be noticed for all the analyzed pellets, compared to the requirements of the standard. This is due to the high nitrogen content of pine sawdust. However, the addition of waste glycerol contributes to a decrease in the nitrogen content in the fuel, so it may have a beneficial effect on the decrease in the NO_x_ concentration during the combustion of fuels with this type of additive.

### 3.2. Results of the Numerical Calculations

**Figure 4:**
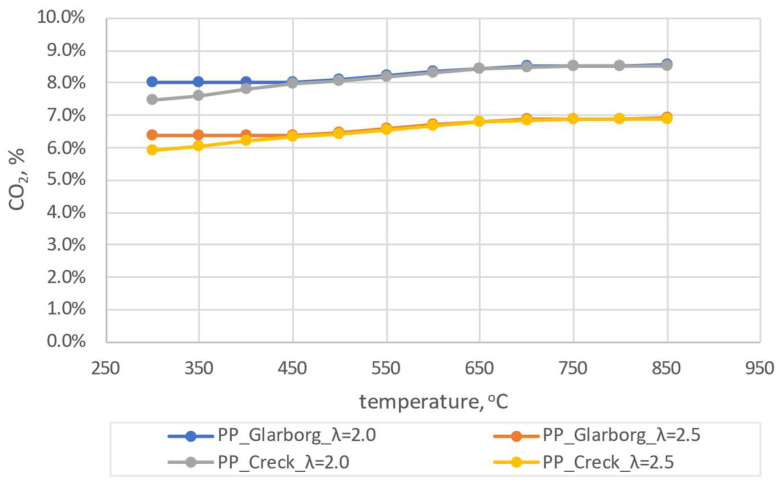
The dependence of CO_2_ shares as a function of temperature for pine pellets–comparison of two mechanism.

**Figure 5:**
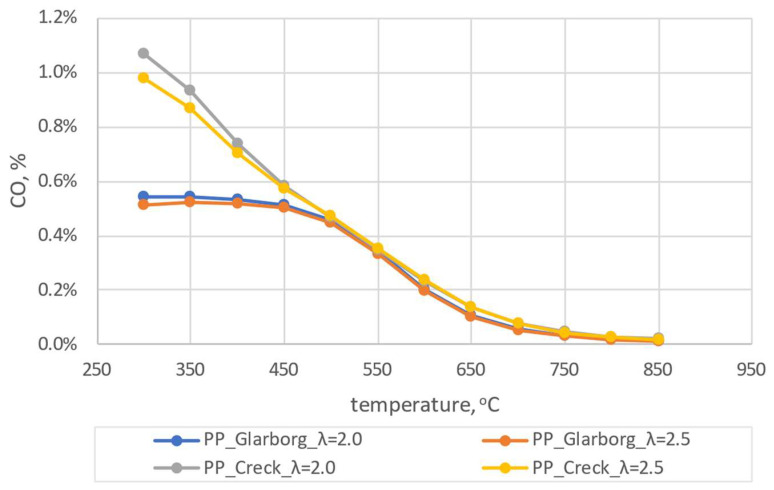
The dependence of CO shares as a function of temperature for pine pellets–comparison of two mechanism.

**Figure 6:**
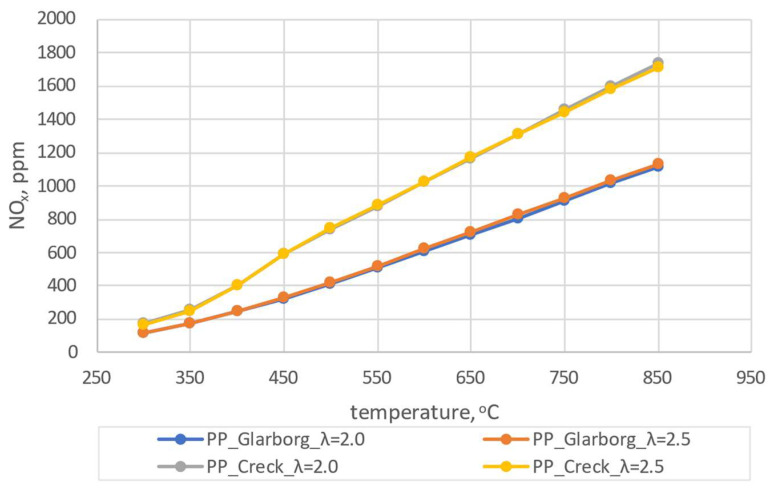
The dependence of NO shares as a function of temperature for pine pellets – comparison of two mechanism.

The results of the initial simulations were carried out for pine pellets (PP). Due to the fact that during the experiment the coefficient of excess combustion air oscillated within λ = 2.0 (± 0.5), numerical simulations were carried out for λ = 2.0 and 2.5. The dependence of the combustion temperature on the concentration of CO_2_, CO and NO_x_ is shown in Figure 4, Figure 5 and Figure 6.

**Figure 7:**
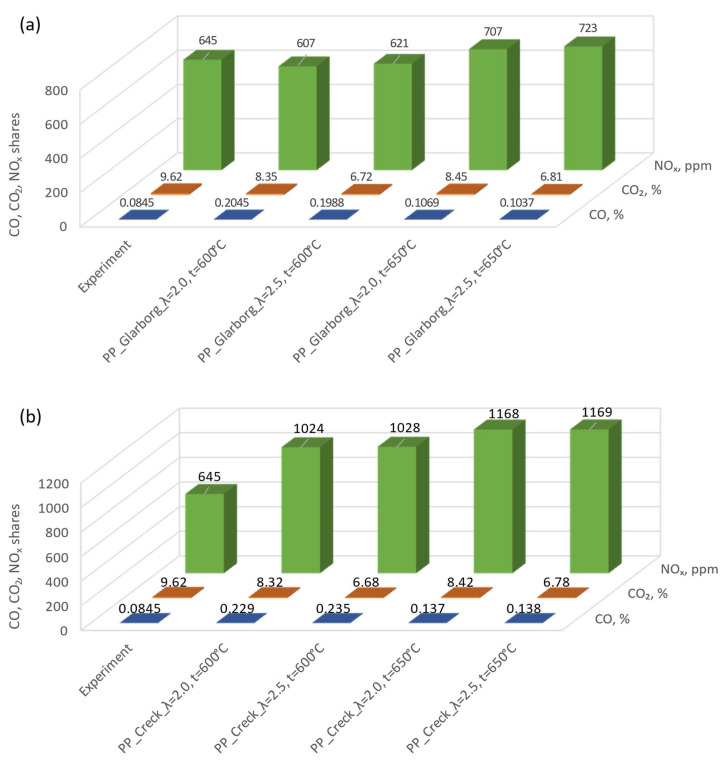
Comparative analysis of experimental and computational results using Glarborg (**a**) and Creck (**b**) mechanisms for pine pellets.

From the analysis presented in Figure 4, Figure 5 and Figure 6, discrepancies can be observed for the two analyzed mechanisms. These discrepancies are observed for CO_2_ and CO at lower temperatures, with the largest ones observed for λ = 2.0 and T = 300 °C and amounting to 0.53% and 0.52%, respectively. In the case of NO_x_ concentration dependence on temperature, the largest discrepancies can be observed for higher temperatures (624 ppm for T = 850 °C and λ = 2.0). For the lowest temperatures considered, these discrepancies are much lower (average 54 ppm). Due to the existing discrepancies, in order to select the mechanism for further consideration, a comparative analysis with experimental studies was carried out. During the combustion of the analyzed pellets, the temperature was close to 600 ± 50 °C and the excess air coefficient was λ = 2.0 ± 0.5. Therefore, the results of numerical simulations obtained for T = 600 °C T = 650 °C, λ = 2.0 and λ = 2.5 were implemented for the comparative analysis. A summary of the results for both mechanisms and experimental results for the pine pellet (PP) is shown in Figure 7.

**Figure 8:**
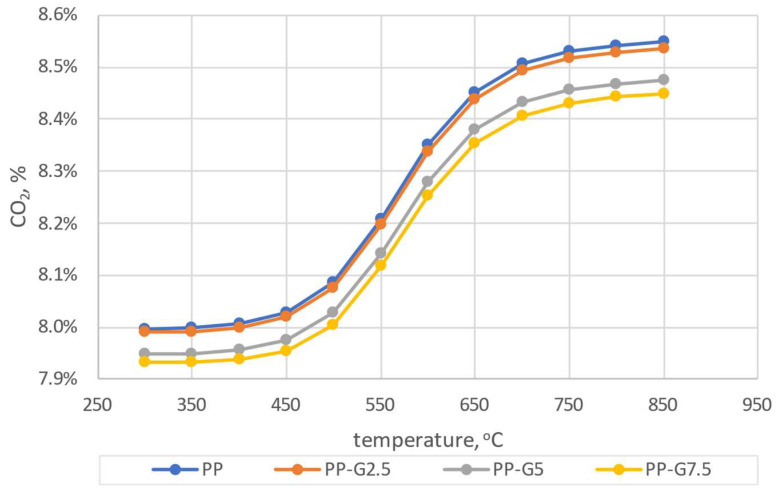
The dependence of CO_2_ shares as a function of temperature.

**Figure 9:**
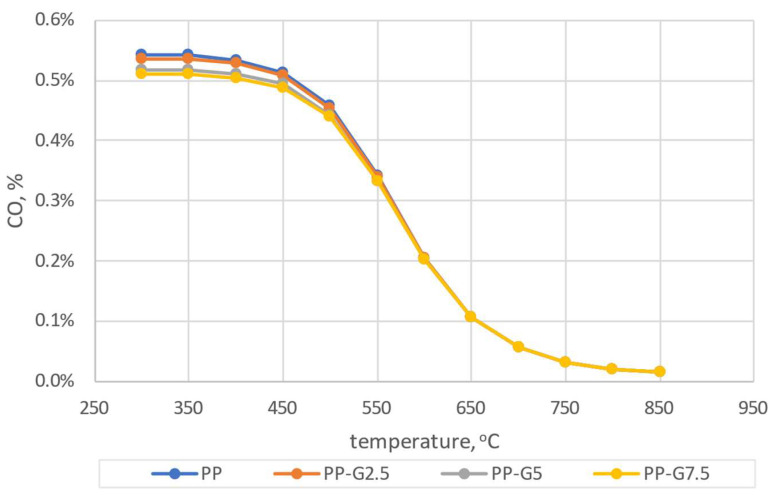
The dependence of CO shares as a function of temperature.

**Figure 10:**
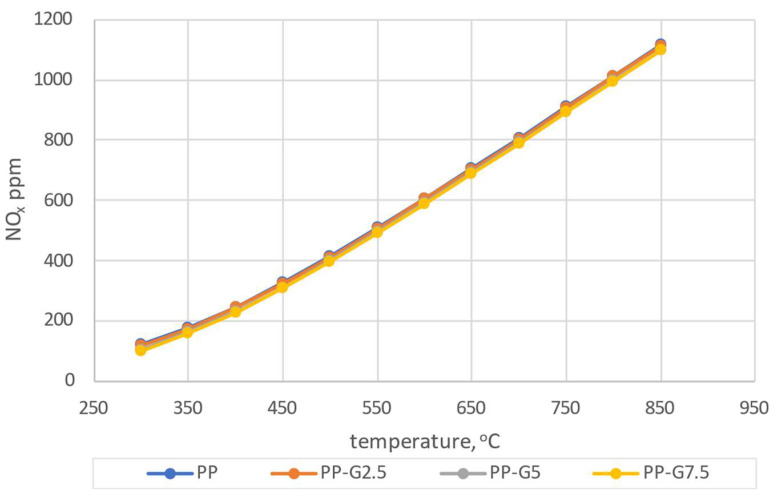
The dependence of NO shares as a function of temperature.

Comparing the results of the numerical simulations with the experimental results, a greater convergence with the calculations carried out using the mechanism developed by Glarborg was found (Figure 7a). In the case of calculations taking into account the Creck mechanism, discrepancies with the experiment of almost 400 ppm can be seen for the NO_x_ concentration. The CO_2_ concentration depends on the coefficient of excess combustion air and shows a similar tendency for both mechanisms. In the case of the CO concentration, there are significant discrepancies between experimental and calculated results. However, in the case of the Glarborg mechanism, these discrepancies are smaller. Such a similarity was also observed in a previous article [59]. Due to the above, the Glarborg mechanism was taken into account for further comparative analyses. Analyzing the results presented in Figure 7a, the greatest convergence can be observed for the model results obtained for T = 650 °C and λ = 2.0, and therefore, these parameters were taken into account for further considerations. Target numerical tests were carried out for four types of analyzed pellets (PP, PP-G2.5, PP-G5 and PP-G7). Simulations were carried out based on the experimental conditions presented in Table 1 and Table 2 (chamber dimensions, fuel consumption, air stream, elemental composition of pellets). Simulations were carried out for the temperature range of 300–850 °C. The results of the dependence of changes in CO_2_, CO and NO_x_ concentrations on temperature for the four considered pellets are shown in Figure 8, Figure 9 and Figure 10.

**Figure 11:**
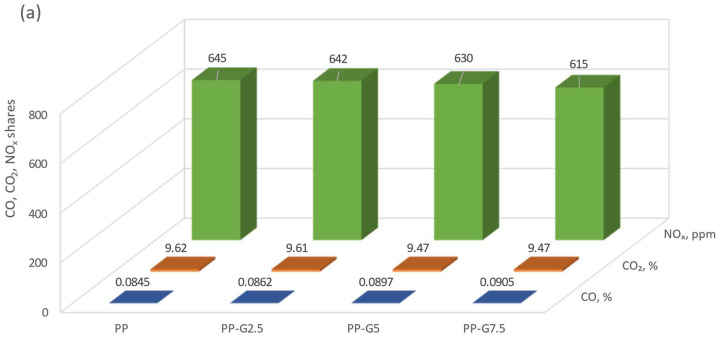

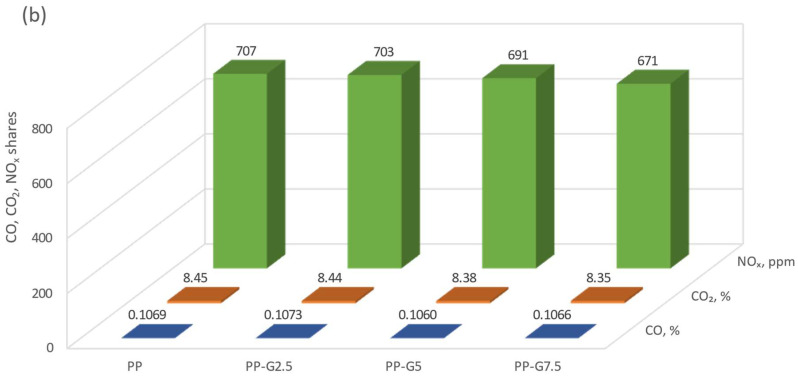
A comparative analysis of the experimental (**a**) and computational (**b**) results for the temperature of 650 °C and λ = 2.0.

It can be concluded from the simulations that the addition of waste glycerol has an effect on reducing the CO_2_ concentration in the entire range of the analyzed temperatures. A similar tendency is noticeable in the case of CO concentration in the lower temperature range, up to about 500 °C. At higher temperatures, the differences resulting from the different additions of glycerol are not so significant. In the case of the analysis of the NO_x_ concentration, no significant deviations depending on the temperature are observed. To illustrate the exact range of concentration differences, taking into account the previous considerations (3.1) and knowing the conditions of the experiment, the results of the simulation for the temperature of 650 °C were taken into account for the comparative analysis (Figure 11).

Comparing the results of experimental data and numerical calculations, one can observe convergent trends for the exhaust gas components in question. With the increase in the share of glycerol in the burned pellets, a decrease in the concentration of CO_2_ and NO_x_ is observed. This beneficial phenomenon results from the lower share of carbon and lack of nitrogen in the waste glycerol, compared to pine wood. The smallest differences between the experiment and the calculation results are visible for NO_x_ and are higher by about 60 ppm in the case of calculations. Both for the calculations and the results of the experiment, it can be observed that the addition of glycerol reduces the NO_x_ concentration in the exhaust gas. While the combustion of pine pellets (PP) only or with a small admixture of waste glycerol (PP-G2.5) does not significantly reduce the NO_x_ concentration, the introduction of 5% and 7.5% glycerol to pellets clearly reduces such a concentration. This favorable phenomenon is due to the lower share of nitrogen in fuels containing glycerol (Table 2). A similar decreasing trend can be observed for the CO_2_ concentration, which, as mentioned earlier, results from a lower share of the carbon element in glycerin waste than in pine wood. From the results of numerical tests, it can be observed that the concentration of CO is practically at the same level, regardless of the type of fuel used. However, the experiment shows that higher amounts of glycerol favor the formation of CO in the exhaust gas, which is a harmful phenomenon. The higher concentrations of CO recorded during the experiment should be explained by the deterioration of combustion conditions along with the increase in the share of glycerol in the pellets. It should be noted that the burner in the boiler is adapted to burn pure wood fuels, therefore the addition of waste glycerol made the operation of the boiler unstable. Larger fluctuations in temperature and the coefficient of excess combustion air were observed. In addition, the 7.5% share of waste glycerol in pine pellets was the maximum that allowed the combustion process to be carried out. Higher shares contributed to the deterioration of fuel parameters, which resulted in spontaneous extinguishing of the boiler. It should be noted that the PSR (Perfectly Stirred Reactor) reactor was used for numerical simulations, which ensured perfect mixing of reagents. Such conditions are not always possible to meet during the experiment. Therefore, the small discrepancies visible between the results of the experiment and the calculations should be explained by the instability of the combustion process.

## 4. Conclusions

In the era of the energy crisis and the limited resources of fossil fuels, it is extremely important to find cheap, alternative, environmentally friendly solutions, the so-called bio-fuels. The role of this type of fuel is to meet expectations, both in terms of ecology and the economy. This article examines the possibility of using waste glycerol as an additive to pine pellets. This solution allows for the cheap management of post-production waste, and it may also contribute to the improvement of selected physicochemical properties of the fuel and thus lowering the emission of harmful combustion products. The experimental and numerical research carried out allowed the following conclusions to be formulated:

− All four types of analyzed pellets meet the requirements of the EN-ISO-17225-2:2014 standard in terms of calorific value, moisture content, ash, bulk density and dimensions The highest values of moisture (4.58%), ash (0.5%) and bulk density (650 kg/m^3^) were observed for PP. The addition of waste glycerol slightly increases the calorific value of the pellet (17.94 MJ/kg for PP-G7.5).
− The higher nitrogen content in relation to the requirements of the standard for all four analyzed pellets results from the high nitrogen content (4.12%) of the base material (pine sawdust).
− The addition of waste glycerol contributes to the reduction in nitrogen and carbon content in the fuel, which has a positive effect on the reduction in NO_x_ and CO_2_ concentrations in exhaust gases. The addition of 7.5% glycerin waste contributes to the reduction in NO_x_ concentrations by 30 ppm and CO_2_ by 0.15%.
− The Glarborg mechanism used in numerical calculations shows a high convergence with the experimental results in comparison to the Creck mechanism (over 400 ppm difference at 650 °C for NO_x_ between the considered mechanisms).
− Difficulties in burning pellets with a 7.5% and higher content of waste glycerol result from adapting the burner to burning wood fuels.
− The obtained test results indicate the legitimacy of further analysis in terms of the efficiency of the combustion of this type of pellet in boilers equipped with an industrial burner.

## Figures and Tables

**Table 1:** Comparison of fuel feed rate and air flow during combustion of pine (PP) and pine–glycerol pellets (PP-G).

Fuel	Fuel Feed Rate, kg/s	Air Flow, m^3^/s	
		λ = 2.0	λ = 2.5
PP	0.000250	0.002322	0.002903
PP-G2.5	0.000272	0.002522	0.003153
PP-G5	0.000292	0.002712	0.003390
PP-G7.5	0.000319	0.002959	0.003698

**Table 2:** A comparison of the selected physicochemical properties and elemental composition for pine pellets without (PP) and with waste glycerol (PP-G2.5, PP-G5, PP-G7.5).

Analyzed Parameters	PP	PP-G2.5	PP-G5	PP-G7.5	EN ISO-17225-2:2014 [74]
*Proximate analysis*					
Moisture, % (±0.1%)	4.58	4.30	4.12	3.76	≤10
Ash, % (±0.1%)	0.50	0.50	0.41	0.40	≤3
*Ultimate analysis*					
C, % (±0.1%)	46.41	46.23	45.82	45.58	-
H, % (±0.1%)	6.45	6.52	6.78	6.86	-
N, % (±0.1%)	4.12	4.09	4.02	4.00	≤1
O, % (bal.)	37.94	38.36	38.85	39.4	-
Diameter, mm (±1 mm)	6	6	6	6	6
Length, mm (±1 mm)	5–15	5–15	5–15	5–15	3.15–40
Calorific value, MJ/kg (±0.1 MJ/kg)	17.63	17.69	17.82	17.94	≥16.5
Bulk density, kg/m^3^ (±10 kg/m^3^)	650	650	620	560	≥500

## Data Availability

The data presented in this study are available on request from the corresponding author.
